# Magnetic circularly polarized luminescence from spin–flip transitions in a molecular ruby†

**DOI:** 10.1039/d4sc04718d

**Published:** 2024-10-01

**Authors:** Alessio Gabbani, Maxime Poncet, Gennaro Pescitelli, Laura Carbonaro, J. Krzystek, Enrique Colacio, Claude Piguet, Francesco Pineider, Lorenzo Di Bari, Juan-Ramón Jiménez, Francesco Zinna

**Affiliations:** a Dipartimento di Chimica e Chimica Industriale, University of Pisa Via Moruzzi 13 56124 Pisa Italy francesco.zinna@unipi.it; b Department of Physics and Astronomy, University of Florence Via Sansone 1 50019 Sesto Fiorentino Italy; c Department of Inorganic and Analytical Chemistry, University of Geneva 30 Quai E. Ansermet CH-1211 Geneva 4 Switzerland; d National High Magnetic Field Laboratory, Florida State University Tallahassee Florida 32310 USA; e Departamento de Química Inorgánica, Facultad de Ciencias, University of Granada, Unidad de Excelencia en Química (UEQ) Avda. Fuente Nueva s/n 18071 Granada Spain jrjimenez@ugr.es

## Abstract

Magnetic circularly polarized luminescence (MCPL), *i.e.* the possibility of generating circularly polarized luminescence in the presence of a magnetic field in achiral or racemic compounds, is a technique of rising interest. Here we show that the far-red spin–flip (SF) transitions of a molecular Cr(iii) complex give intense MCD (magnetic circular dichroism) and in particular MCPL (*g*_MCPL_ up to 6.3 × 10^−3^ T^−1^) even at magnetic fields as low as 0.4 T. Cr(iii) doublet states and SF emission are nowadays the object of many investigations, as they may open the way to several applications. Due to their nature, such transitions can be conveniently addressed by MCPL, which strongly depends on the zero field splitting and Zeeman splitting of the involved states. Despite the complexity of the nature of such states and the related photophysics, the obtained MCPL data can be rationalized consistently with the information recovered with more established techniques, such as HFEPR (high-frequency and -field electron paramagnetic resonance). We anticipate that emissive molecular Cr(iii) species may be useful in magneto-optical devices, such as magnetic CP-OLEDs.

## Abstract 2

Magnetic circularly polarized luminescence of spin–flip transitions of a Cr(iii) complex is measured at low field.

## Graphical abstract

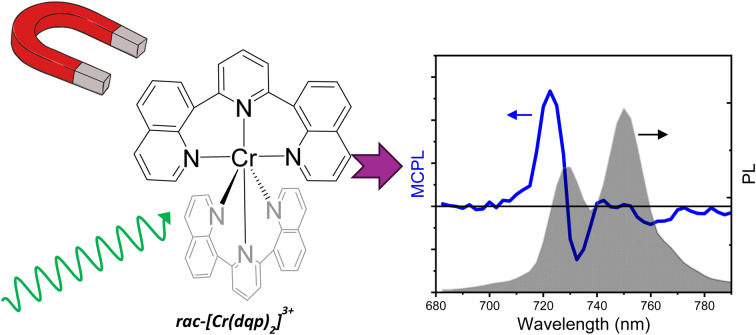

## Introduction

Molecular complexes based on d-metals offer a diverse and intriguing photophysics,^1^ with applications ranging from photocatalysis,^2^ optoelectronics,^3,4^ imaging^5,6^ and photodynamic therapy.^7–9^ To understand the often non-trivial photophysics at play, the use of less common spectroscopic techniques may be beneficial. In turn, this is necessary to exploit the full potential of those systems and to give indications for a rational design of the ligands and complexes.

**Fig. 1:**
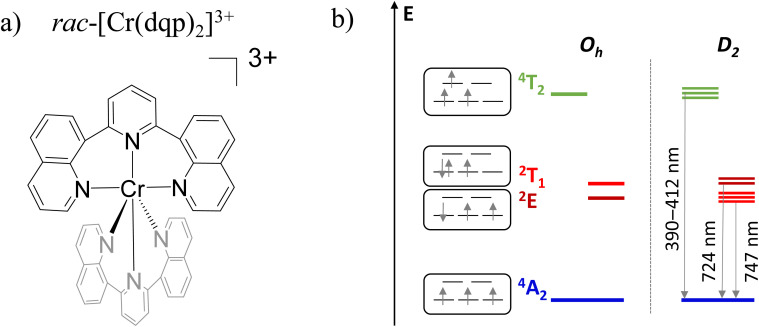
(a) Structure of the complex. (b) Electronic states of [Cr(dqp)_2_]^3+^ in an ideal octahedral (*O*_h_) and *D*_2_ geometry; note the ^2^T_1_/^2^E level inversion from *O*_h_ to *D*_2_ geometry.

A particularly interesting case is observed when metal-centred excited states differ only by spin configuration with respect to the ground state.^10^ Such configurations are called spin–flip (SF) states and they may display sharp phosphorescent transitions (SF-transitions), forbidden by electric transition moment, with lifetimes up to a millisecond. SF luminescence was observed in the case of V(ii)/V(iii),^11–13^ Mn(iv),^14,15^ Mo(iii),^16,17^ Re(iv),^17^ and in particular, remarkable results in terms of emission efficiency were obtained in the case of (pseudo)octahedral Cr(iii) complexes.^18–20^ Such complexes show luminescence associated with the doublet states ^2^T_1_/^2^E (Fig. 1) with quantum yields up to 30% with narrow bands in the far red or near infrared region.^18^ These features, reminiscent of those of the ruby gemstone, can be obtained in a molecular compound with octahedral-like geometry (i) to avoid excited state distortions and (ii) to induce strong ligand field splitting, needed to shift the ^4^T_2_ states toward higher energy thus preventing deactivation of the SF states due to back intersystem crossing (BISC).^19^ Complexes featuring SF states may be exploited as optical probes for oxygen,^20^ pressure^21^ and temperature,^22^ photocatalysis^23–25^ and photocathodic solar cells.^19,26^

Concerning the luminescence activity associated with the SF transitions, the most promising results have been achieved so far by employing two families of Cr(iii) complexes: [Cr(ddpd)_2_]^3+^ (ddpd = *N*,*N*′-dimethyl-*N*,*N*′′-dipyridin-2-ylpyridine-2,6-diamine)^18,27,28^ and [Cr(dqp)_2_]^3+^ (dqp = 2,6-di(quinolin-8-yl)pyridine).^29,30^ In those cases, the first coordination sphere is roughly octahedral, but the arrangement of the tridentate organic ligand around the Cr-center defines a *λ*/*δ* chirality in an overall *D*_2_ geometry.^31^ Thanks to the electric dipole forbidden nature of the SF transition, in enantiopure form, such compounds display highly circularly polarized luminescence (CPL), with dissymmetry factors (*g*_lum_) on the order of 10^−1^.^30–34^ Such values are comparable with those obtained for the f–f transitions of chiral lanthanide(iii) complexes,^35–39^ but Cr offers the advantage of being cheaper, kinetically inert and more abundant than lanthanides.^40^

A different technique to study the circular polarization of the emitted light is magnetic CPL (MCPL), where the physical origin of the CP emission is not the chirality of the material, but the effect is triggered by the application of an external magnetic field. This technique belongs to the family of magneto-optical spectroscopies, along with the more common Faraday rotation and magnetic circular dichroism (MCD).^41–43^ In a MCPL experiment, the circular polarization of the luminescence is studied, when the sample is placed under a magnetic field collinear with the emission direction, and excited with unpolarized light.^44,45^ Unlike CPL, MCPL may be displayed by both chiral and achiral luminescent systems and it does not depend on the enantiomer chirality. Indeed, CPL and MCPL follow very different selection rules. CPL is gauged by the scalar product *m*_ng_·*μ*_gn_, where *m* and *μ* are the magnetic and electric transition moments between the ground state g and excited state(s) n.^46^ On the other hand, several mechanisms can lead to MCPL. Relatively strong signals are predicted in the case of orbital or spin degenerate ground or excited states, where the degeneracy is removed by the magnetic field due to the Zeeman effect. In these cases, the MCPL signal depends on *m*_gg_*μ*_ng_*μ*_gn_ and *m*_nn_*μ*_ng_*μ*_gn_ products (*m*_gg_ and *m*_nn_ are the static magnetic dipole moments of the ground and excited state), for a degenerate ground or excited state respectively.^47,48^ Those expressions are associated to the so-called Faraday A- and C-terms.^44,47,49,50^ Emitting compounds characterized by a strong spin–orbit coupling, which allows for a significant mixing of states, are good candidates for magneto-optical spectroscopies, including MCPL.

MCPL has been thus studied in the case of f–f transitions(iii) of lanthanide complexes,^51–54^ d-metals (such as Ru(ii), Ir(iii) and Pt(ii) complexes),^55–58^ organic and metallo-organic compounds,^59–69^ as well as other metal-based materials.^70–72^ MCPL and MCD were also reported in early studies of Cr(iii) inorganic structures.^73,74^ MCD was also used to study far red/near infrared (NIR) transitions in the case of Ir(iii) or Pt(ii) complexes,^75,76^ where their observation through emission, and thus MCPL, is challenging. On the other hand, emissive Cr(iii) compounds are potentially a more suitable platform to address metal transitions through MCPL. On a fundamental level, an analysis of the MCPL spectrum can elucidate the nature of the excited and ground states, Zeeman effects, *etc.*, and along with other techniques can help to understand the full picture of the photophysics of a complex system.^44^ Moreover, MCPL-active compounds have recently found applications in OLEDs able to emit circularly polarized electroluminescence in a magnetic field (MCP-OLEDs),^77–81^ therefore there is also a practical interest in unveiling different types of emitters endowed with significant MCPL.

In the following, we investigate the racemic [Cr(dqp)_2_](PF_6_)_3_ material by MCPL and MCD (Fig. 1b). As introduced above, [Cr(dqp)_2_]^3+^ is one of the archetypes of a molecular ruby and the same concept shown here may be applied to similar systems.

## Results and discussion

**Fig. 2:**
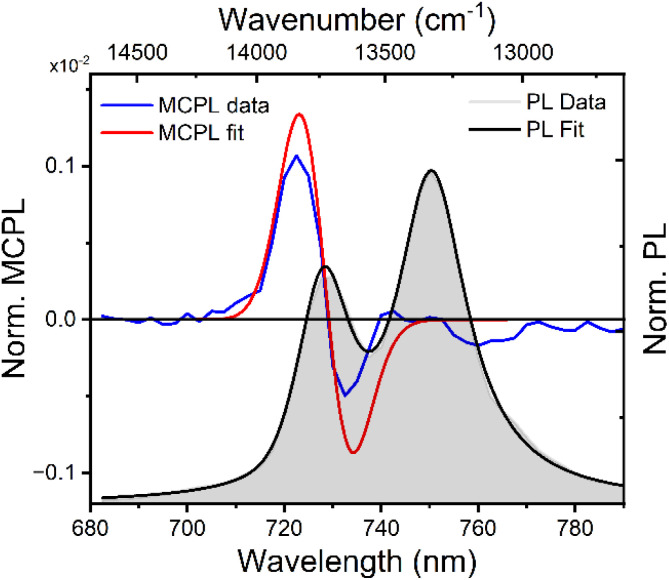
PL and MCPL at 0.4 T, normalized for the PL maximum, of the Cr^3+^ complex dissolved in deareated acetonitrile, along with the corresponding fittings according to the rigid-shift model (see the text).

Optical and magneto-optical studies reported here were performed on deaerated acetonitrile solutions of the racemic compound ([Cr(dqp)_2_](PF_6_)_3_). The photoluminescence (PL) spectrum of [Cr(dqp)_2_]^3+^ shows two main emission bands centred at 750 and 729 nm (Fig. 2), associated to the SF transitions (^2^E/^2^T_1_ → ^4^A_2_) from the sublevels of the doublet excited states (see Fig. 1a) to the ground state. The lower energy band appears more intense than the higher energy one by a factor of 1.53 at 300 K, due to a higher Boltzmann population with an energy gap of ≈420 cm^−1^ between the two bands.^27^

We measured the MCPL emission at 300 K under a magnetic field of ±0.4 T generated by a permanent magnet, exciting the sample at 365 nm (see the ESI and Fig. S1† for the details of the measurement set-up). In these conditions, a relatively strong and slightly asymmetric bisignate MCPL band was observed corresponding to the higher energy doublet transition (Fig. 2). Such band has a cross-over point at 725 nm, matching the maximum of the corresponding photoluminescence band. This derivative-like shape is consistent with a signal originating from Zeeman-split states (see below). The MCPL strength can be quantified by a magnetic field (*H*) normalized dissymmetry factor (*g*_MCPL_), defined as:1
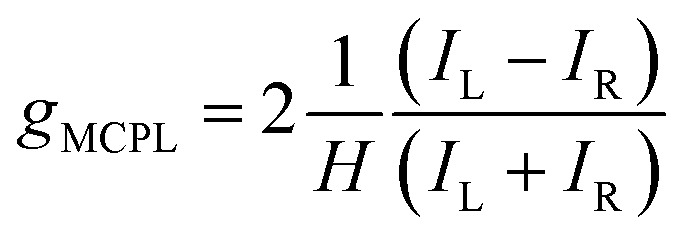
where *I*_L_ and *I*_R_ are the left and right circularly polarized components of the emission under the magnetic field. In our case, we found a *g*_MCPL_ of 6.3 × 10^−3^ T^−1^ at 722 nm and −2.1 × 10^−3^ T^−1^ at 733 nm. These values are in line with those obtained for other d or f-metal complexes.^52,53,55,82^ A much weaker (≈−0.9 × 10^−3^ T^−1^) band around 750 nm associated the lower energy SF transition was also observed. As expected, roughly mirror image MCPL spectra were obtained for +/− magnetic field (Fig. S3a†), by reversing the orientation of the permanent magnet. In Fig. 2, we report the MCPL as the semi-difference of the polarized signal *S* obtained under positive (*S*(+)) and negative (*S*(−)) *H*, as:2
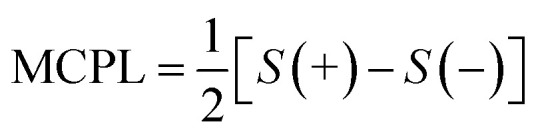


Positive and negative fields are here defined as parallel and anti-parallel to the *k*-vector, *i.e.* the propagating direction of light. Baseline effects and possible artefact signals due to photoselection cause some deviation from the mirror image relationship expected for spectra obtained under opposite magnetic field, especially in the case of small signals (Fig. S3a†). The data treated according to eqn (2) ensures that such artefacts, which are magnetic field-independent, are eliminated and only the true MCPL is recovered (Fig. S3b†).

**Fig. 3:**
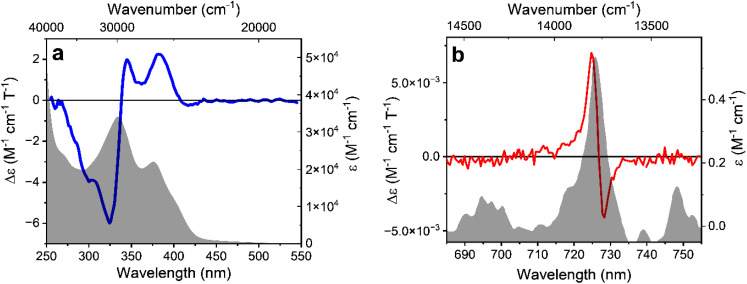
MCD and extinction spectra of an acetonitrile solution of [Cr(dqp)_2_]^3+^, for the higher energy region (a) and for the SF transition region (b). The data are normalized for concentration, optical path and applied magnetic field.

The same SF transitions were also studied in absorption through MCD, on a concentrated solution of the complex under ±1.4 T magnetic field (Fig. 3b), generated by an electromagnet. A derivative-like signal was observed associated to the higher energy doublet transition, similar to the corresponding MCPL band. No significant MCD corresponding to the lower energy SF transition was detected. Similarly to the MCPL, MCD dissymmetry factor (*g*_MCD_) can be defined as:3
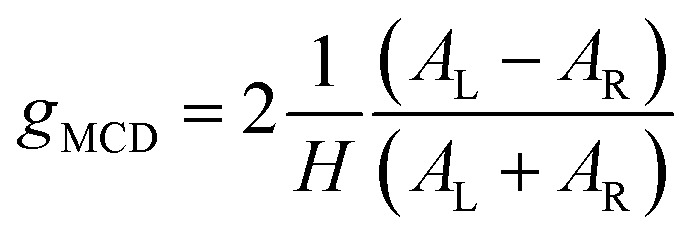
with *A*_L_ (*A*_R_) the absorbance of left (right) circularly polarized light. The *g*_abs_ were estimated approximately +1.7 and −1.4 × 10^−2^ T^−1^ at 725 and 728 nm respectively. Such values are 2 orders of magnitude higher than those observed for the MCD bands between 500 and 250 nm (Fig. 3a), associated with the ligand-centred (LC) and ligand-to-metal charge-transfer transitions (*g*_MCD_ ≈ 1 × 10^−4^ T^−1^). This confirms that magneto-optical techniques are excellent tools to study SF transitions. Such studies are particularly challenging in standard absorption spectroscopy experiments due to the very weak extinction coefficient of SF transitions (below 1 M^−1^ cm^−1^, see Fig. 3b), which requires high concentrations and deconvolution from the tail of the high energy absorption transitions, making it difficult to distinguish the extinction peaks from the instrumental noise. On the other hand, we show here that MCD experiments can give signals well above the instrumental noise, without the need for deconvolution procedures. As expected, the MCD signal intensity was found to be linear with the applied field *H* (Fig. S4†). The overall similarity of the MCD and MCPL shape and signature is consistent with minor structural differences between the ground state and the doublet states of Cr(iii). Indeed, SF states have a nested nature and therefore they are expected to be only weakly distorted with respect to the ground state.^10,23^

**Fig. 4:**
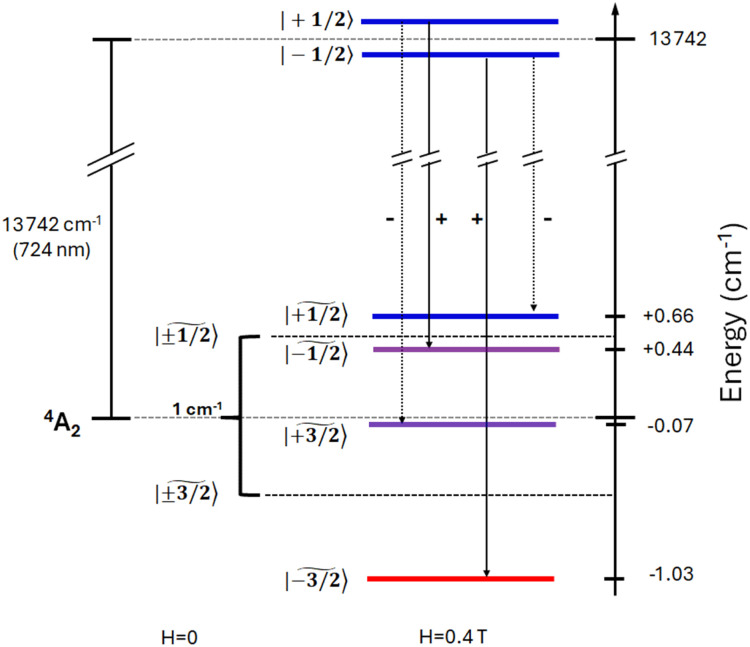
ZFS- and Zeeman-split levels at 0.4 T corresponding to the main MCPL bands. The MCPL transitions are represented by the dotted arrows along with the expected sign. The + and − symbols indicate left and right circularly polarized emissive transitions respectively. The energies of ground state sublevels (for *H*_0_∥*z*) are obtained through HFEPR analysis (see Fig. 5 and Table S3†). As considerable mixing occurs (see Table S3†), the predominant character of the *M*_s_ state is indicated by a ∼ symbol. Purple colour indicates strongly mixed states.

To rationalize these results, it is worth analysing the states involved in the SF transitions giving origin to the MCD and MCPL spectra. In the complex, the presence of the helically twisted tridentate dqp suppresses any symmetry plane and lowers the overall symmetry to *D*_2_. In such symmetry, the orbital degeneracy of the excited states is removed, and ^2^T_1_ and ^2^E states are split into 3 and 2 components respectively (Fig. 1a), the main MCPL signal at 729 nm is associated with the lower component of the doubly degenerate ^2^E state. A small contribution to the PL, giving non-significant MCPL, is observed at approximately 710 nm, possibly stemming from the higher energy component of the ^2^E state (Fig. S2†). Moreover, even in the absence of an external field, the orbitally-nondegenerate quartet ground state (^4^A_2_) is split by the zero-field splitting (ZFS) into two Kramers doublets (KD) |±1/2〉 and |±3/2〉 (Fig. 4).

To extract the ZFS and the corresponding spin Hamiltonian *D* and *E* parameters, we followed the method proposed by van Slageren *et al.* (see ESI†).^83^ As the first step, the energies corresponding to the 3 ^4^A_2_ → ^4^T_2_ term-to-term transitions were determined by a phenomenological deconvolution of the 360–500 nm region of the MCD spectrum (Fig. S5†). With this procedure, we identified the following energies, centred approximately at 24 220, 24 922 and 25 630 cm^−1^ (Table S1†). From these values (see ESI† for the formulae in a *D*_2_ geometry), we calculated *D* and *E* parameters as approximately 0.51 and 0.16 cm^−1^, respectively, with a rhombicity factor *E*/*D* = 0.32, close to the maximum (1/3). The corresponding ZFS was calculated to be ≈1.1 cm^−1^ as 
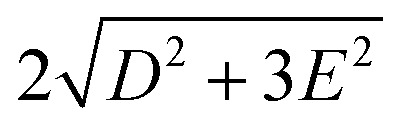
 (see Fig. 4). Despite the large error of the method (see for instance the standard errors on the fitting coefficient in Table S1†), the values are comparable with those found for the analogue complex [Cr(ddpd)_2_]^3+^,^83^ and the values found by HFEPR (high-frequency and -field electron paramagnetic resonance) on our complex (see below).

We now focus our analysis on the MCPL and PL spectra at the NIR SF transitions. In the presence of an external magnetic field, the degeneracy of the KDs of the quartet ground states is removed by Zeeman effect giving 4 spin states: ideally |+1/2〉, |−1/2〉, |+3/2〉 and |−3/2〉. Similarly, the doublet excited states split into |+1/2〉 and |−1/2〉 states.

In the following, we focus only on the higher energy SF band around 729 nm, producing the main signal. In a reasonably simplified scheme, we consider only the transitions among Zeeman levels with Δ*M*_s_ = ±1.^84^ A Δ*M*_s_ > 0 corresponds to a positive MCPL transition and *vice versa*, therefore a total of two closely spaced positive and two negative MCPL contributions are expected (Fig. 4).

When the bands are separated by an energy much smaller than the bandwidth, the spectral features can be conveniently modelled by using the so-called rigid-shift approximation. This method is usually applied to model A- and C-Faraday terms in MCD.^43,85,86^ Within this model, PL and MCPL spectra are fitted simultaneously using a home-built MATLAB code, employing fitting functions that hold shared parameters. The emission is fitted with a bell-shaped function centred on the energy barycentre (unsplit levels), while the MCPL is fitted with equal but opposite functions, displaced by the small field-induced splitting (see ESI and Fig. S6†). All the functions, used for both the PL and MCPL, share the same shape and bandwidth, and are therefore determined simultaneously in the fitting. To carry out this procedure, we used the four energy levels of the ^4^A_2_ state determined by HFEPR at 0.4 T (see below), as fixed parameters. The fitting obtained through this model, by using pseudo-Voigt functions, closely retraces the experimental PL and MCPL data (Fig. 2 and ESI†). Similar results were obtained using purely Gaussian or Lorentzian functions, but pseudo-Voigt functions retrace better the PL and MCPL line shapes (Fig. S7 and Table S2†). The overestimation of the model with respect to the experimental MCPL data may be due to the fact that, in the case of ZFS with *E* ≠ 0, the four states associated with ^4^A_2_ cannot be described by a pure *M*_s_ quantum number as they are significantly mixed (see below). This would therefore impact the underlying assumption that each transition is completely circularly polarized. Notice that the lifetime of the excited state being sufficiently long (*τ*_obs_ = 1.2 ms),^33^ the population of its Zeeman levels follows Boltzmann distribution. The Zeeman splitting being much smaller than room temperature thermal energy (207 cm^−1^ at 298 K), the Zeeman levels of the doublet excited states are almost equally populated, as 1 − (Δ*E*/*k*_b_*T*) ≈ 0.998. Fig. 4 quantitatively summarizes the fine structure of the electronic level involved in the MCPL emission of the main band.

To corroborate the analysis and demonstrate the consistency of our approach, we performed magnetometry and EPR characterization. DC magnetometry, studied in the 2–300 K temperature range, confirms a quartet ground state (^4^A_2_, see ESI and Fig. S8†). Saturation magnetization at 2 K is consistent with what expected for isolated Cr(iii) cations with *g* = 2 and *S* = 3/2. Upon cooling, the *χ*_M_*T* product remains almost constant until about 10 K and then sharply decreases to reach a value of 1.78 cm^3^ mol^−1^ K at 2 K. This decrease is due to the ZFS and Zeeman interactions. The simultaneous fitting of the susceptibility and magnetization data with the ZFS Hamiltonian using the PHI software^87^ leads to a |*D*| value of 0.72 cm^−1^, which is rather consistent with the values extracted from MCD and HFEPR spectroscopies.

**Fig. 5:**
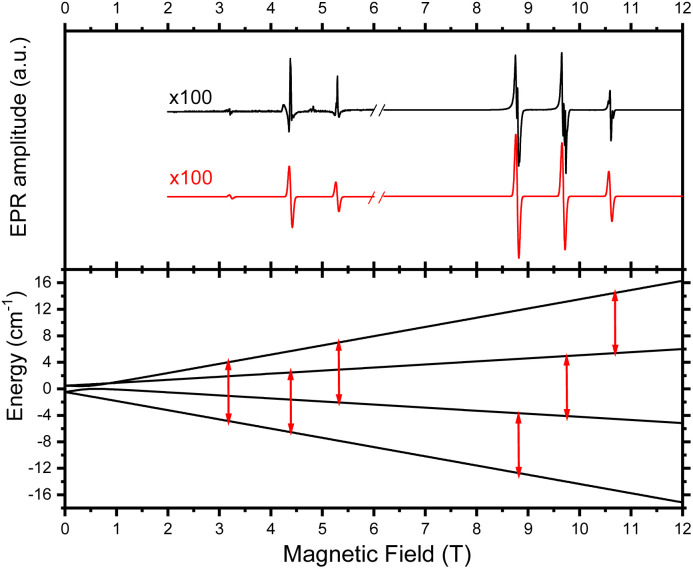
Top: EPR spectrum of a polycrystalline sample containing the Cr(iii) compound at 30 K and 270 GHz (black trace) accompanied by its simulation (red trace) using the following spin Hamiltonian parameters: *S* = 3/2, *D* = 0.42 cm^−1^, *E* = 0.14 cm^−1^ (*E*/*D* = 0.33, maximum rhombicity limit), *g*_iso_ = 1.99. The simulation assumed a single crystal oriented with the *z*-axis of the ZFS tensor parallel to the magnetic field *H*_0_. Bottom: representation of the energy levels for a *S* = 3/2 spin state at 270 GHz. The HFEPR transitions between the spin sublevels are marked with red arrows.

To confirm the determined *D* and *E* values from MCD measurements, the Cr(iii) compound was studied by HFEPR spectroscopy.^88^ The shape and amplitude of the low-temperature (30 K) spectra of the powder sample “as is” (*i.e.* unconstrained) strongly suggest field-induced alignment (*i.e.* torquing) of the crystallites.^89^ Indeed, the resulting spectra could be very well simulated as originating from a single crystal oriented with the *z*-axis of the ZFS tensor parallel to the magnetic field *H*_0_ (Fig. 5). At 270 GHz, the three dominating peaks between 8 and 12 T represent the allowed Δ*M*_s_ = ±1 transitions between the spin sublevels of the *S* = 3/2 spin state of Cr(iii). The weak peaks in the 3–6 T range are the nominally forbidden Δ*M*_s_ = ±2 and ±3 transitions. The structure visible on the peaks is an artefact that can be attributed to imperfect field alignment and is not simulated.

In order to extract the full set of frequency-independent spin Hamiltonian parameters, the sample was constrained using *n*-eicosane and pressed into a pellet (Fig. S9†). The resulting spectrum is accompanied by a simulation, this time assuming a powder distribution of the crystallites in space. The simulation parameters are modified relative to those used above to account for the slight asymmetry of the central line: *D* = 0.43 cm^−1^, *E* = 0.14 cm^−1^ (*E*/*D* = 0.325), *g*_*x*_ = 1.99, *g*_*y*,*z*_ = 1.98.

To finalize the values of frequency-independent spin Hamiltonian parameters, we built a two-dimensional map of turning points in pellet spectra and applied the tunable-frequency methodology^90^ by fitting the parameters simultaneously to that map. This resulted in the following values: *D* = −0.436(7) cm^−1^, *E* = −0.144(7) cm^−1^ (*E*/*D* = 0.31), *g*_iso_ = 1.980(4) (Fig. S10†). The negative sign of *D* reproduced single-frequency spectra better than a positive value.

Altogether, these results are in good agreement with the ones that we found by MCPL and MCD experiments. Finally, the nature of the *M*_s_ states associated with ^4^A_2_ was evaluated by calculating the mixing coefficients at 0.4 T (Tables S3 and S4, see also Fig. S11† for an expansion of HFEPR levels at low field). The coefficients show (in the case of *H*_0_∥*z*, see Table S3†) a strong mixing between two inner |+3/2〉 and |−1/2〉 levels, responsible for the avoided level crossing near 1 T visible in Fig. 5 (bottom), while the outer levels retain mostly their |+1/2〉 and |−3/2〉 character.

## Conclusions

Beyond the many areas of interest in Cr(iii) SF transitions of the molecular ruby [Cr(dqp)_2_]^3+^, we show here that they also display a strong MCPL activity. MCD and MCPL techniques are here applied to elucidate the fine structure of the levels involved in the SF transitions. In particular, the analysis of SF transition through MCPL was consistent with the energies found with the more established EPR spectroscopy. Such possibilities offered by magneto-optical techniques can be exploited to gather more insight into the photophysics of SF transitions in related cases, *e.g.* in view of optical read-out of molecular qubits. Moreover, it opens up new opportunities such as applications in magneto-optical and magnetoelectronic devices.

## Data availability

The data supporting this article have been included as part of the ESI.†

## Author contributions

Conceptualization: F. Z.; data curation: A. G., G. P., F. P., J. R. J., F. Z.; formal analysis: A. G., F. Z.; resources: M. P., C. P., J. R. J.; funding acquisition: L. D. B., C. P., F. P., J. R. J.; investigation: A. G., L. C., E. C., J. K., F. Z. methodology: F. Z., G. P. writing – original draft: A. G., J. R. J., F. Z.; writing – review & editing: all authors.

## Conflicts of interest

There are no conflicts to declare.

## Supplementary Material

SC-OLF-D4SC04718D-s001
