# Supplementary material for: Magnetic circularly polarized luminescence from spin–flip transitions in a molecular ruby
Source: Chem Sci. 2024 Oct 1;15(41):17217–23. doi: 10.1039/d4sc04718d (PMC11443232; doi:10.1039/d4sc04718d)
Supplement: SC-015-D4SC04718D-s001 [file SC-015-D4SC04718D-s001.pdf]

Supplementary Information (SI) for Chemical Science.
This journal is © The Royal Society of Chemistry 2024

Supporting information

Magnetic circularly polarized luminescence from spin flip transitions in a molecular ruby

Alessio Gabbani,[a,b] Maxime Poncet,[c] Gennaro Pescitelli,[a] Laura Carbonaro,[a] J. Krzystek,[d] Enrique Colacio,[e] Claude Piguet,[c] Francesco Pineider,[a,b] Lorenzo Di Bari,[a] Juan-Ramón Jiménez*,[e] Francesco Zinna*[a]

[a] Dipartimento di Chimica e Chimica Industriale, University of Pisa, via Moruzzi 13, 56124, Pisa, Italy
[b] Department of Physics and Astronomy, University of Florence, via Sansone 1, 50019, Sesto Fiorentino, Italy
[c] Department of Inorganic and Analytical Chemistry, University of Geneva, 30 quai E. Ansermet, CH-1211 Geneva 4, Switzerland
[d] National High Magnetic Field Laboratory, Florida State University, Tallahassee, Florida 32310, USA
[e] Departamento de Química Inorgánica, Facultad de Ciencias, Universidad de Granada and Unidad de Excelencia en Química (UEQ), Avda. Fuente Nueva s/n, 18071, Granada, Spain.
E-mail:
jrjimenez@ugr.es, francesco.zinna@unipi.it

Complex Preparation

The $[Cr(dqp)_2](PF_6)_3$ (dqp = 2,6-di(quinolin-8- yl)pyridine) has been prepared according to a published method.[1]

Instrumentation

MCD measurements were carried out using an in-house built, lab-scale instrument equipped with a 1.4 T electromagnet. Light emitted by a 300 W Xe arc lamp (Newport) is monochromated (Newport Oriel Cornerstone 260) and chopped at 440 Hz. Polarization modulation at 47 kHz between LCP and RCP is obtained with a Glan-Thompson polarizer coupled to a photoelastic modulator (Hinds Instruments PEM 100) set to $\lambda/4$ retardation. The beam is then directed through the bore of an electromagnet (Buckley Systems Ltd. GMW model 3470) where the sample is placed. Light is collected by a photomultiplier tube (Hamamatsu R376). After transimpedance amplification, the output voltage of the detector is fed to two lock-in amplifiers, one referenced to the polarization modulation frequency (Stanford Research Systems SR850) and one to the light chopping frequency (Signal Recovery 7280); the ratio of the two signals is taken as the differential absorption (dichroism) ΔA. MCD signal is then obtained by the semi difference between the signals obtained at +1.4 T and -1.4 T. Calibration of the MCD signal is carried out against an aqueous solution of $[Fe(CN)_6]^{3+}$ of known concentration. The MCD spectra of the Cr^{3+} complex were recorded in CH_3CN solution in a 1 mm optical path cuvette. The concentration was $1.67 \cdot 10^{-4}$ M and $2.57 \cdot 10^{-2}$ M for the high and low energy region respectively. Given the slits used (0.6 mm) and the grating employed in the monochromator, the spectral resolution was 1.9 nm. MCD vs field plots in the +1.4/-1.4 T range were also acquired by regulating the current passing in the coils of the electromagnet. The strength of the magnetic field was measured with a Hall probe.

MCPL measurements were carried out with an in-house built set-up mounted on an open optical bench (Figure S1). The sample is placed in front of 0.4 T NdFeB permanent magnet, and it is excited with a 365 nm LED (M365D1, Thorlabs) with a 0° geometry, using a dichroic mirror (cutoff 400 nm). The collected light is passed through a photoelastic modulator (Hinds Instruments PEM 100) set to $\lambda/4$ retardation modulated at 50 kHz, coupled with a Glan-Thompson polarizer. Ambient light is filtered out using a chopper. The light emitted by the sample then passes through a monochromator (Oriel Cornerstone 130) and is detected by a mutlialkali photomultiplier tube (Hamamatsu R376). The monochromator slits were set to 0.7 mm (spectral resolution of 9 nm), as this was found to be a good compromise between having enough light at the detector while ensuring enough spectral resolution to distinguish the two SF transitions (Figure S1). The voltage of the photomiultiplier tube used was 650 V. After transimpedance amplification, the output voltage of the detector is fed to two lock-in amplifiers, one referenced to the polarization modulation frequency (MFLI, Zurich Instruments) and one to the light chopping frequency (Stanford Research Systems SR850). The PL and MCPL spectra are acquired simultaneously by using a LabVIEW routine. The spectra were recorded in a 2 mM CH_3CN solution, in Ar atmosphere, using a rotaflow 1 mm cuvette and deaerated CH_3CN as solvent. 9 spectra for each field (+0.4 and -0.4 T) were acquired and averaged. The excitation LED was powered with a current of 250 mA. The resulting excitation optical power was 48 mW, as measured with a power meter.

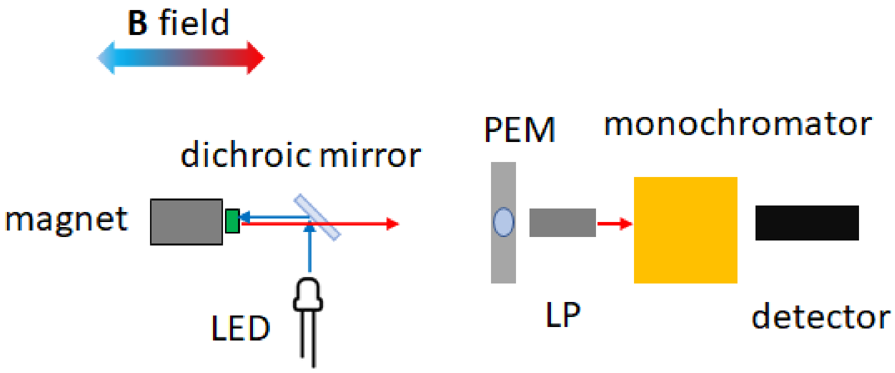

Figure S1. Simplified scheme of the set up employed for MCPL measurements.

High-frequency and -field EPR (HFEPR) spectra were recorded at the National High Magnetic Field Laboratory on 49 mg of polycrystalline sample either loose (unconstrained) or pressed into a pellet with *n*-eicosane. A homodyne spectrometer at the EMR Facility associated with a 15/17-T superconducting magnet is described in bibliography[2] with a modification of using a Virginia Diodes (VDI, Charlottesville, VA, USA) chain operating in the 48 – 540 GHz frequency range. Detection was provided with an InSb hot electron bolometer (QMC Ltd., Cardiff, UK). The magnetic field was modulated at 50 kHz for detection purposes. A Stanford Research Systems SR830 lock-in amplifier converted the modulated signal to *dc* voltage.

Variable-temperature (2−300 K) magnetic susceptibility measurements were carried out on polycrystalline samples under an applied field of 0.3 T using a DynaCool PPMS-9 physical measurement equipment. The magnetic susceptibility values were corrected from the diamagnetism of the molecular constituents and of the sample holder.

Additional figures

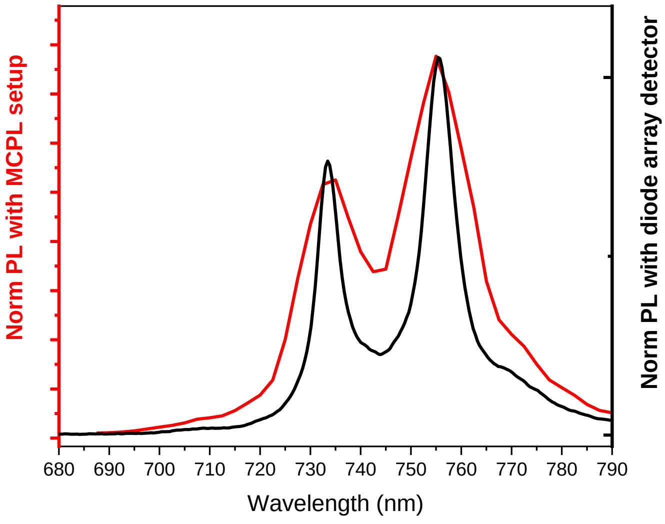

Figure S2: Comparison between the PL measured with the MCPL setup with a spectral resolution of 9 nm, and the one measured with an array detector having 2 nm of spectral resolution (Optosky ATP2000P Modular spectrometer, Crisel Instruments).

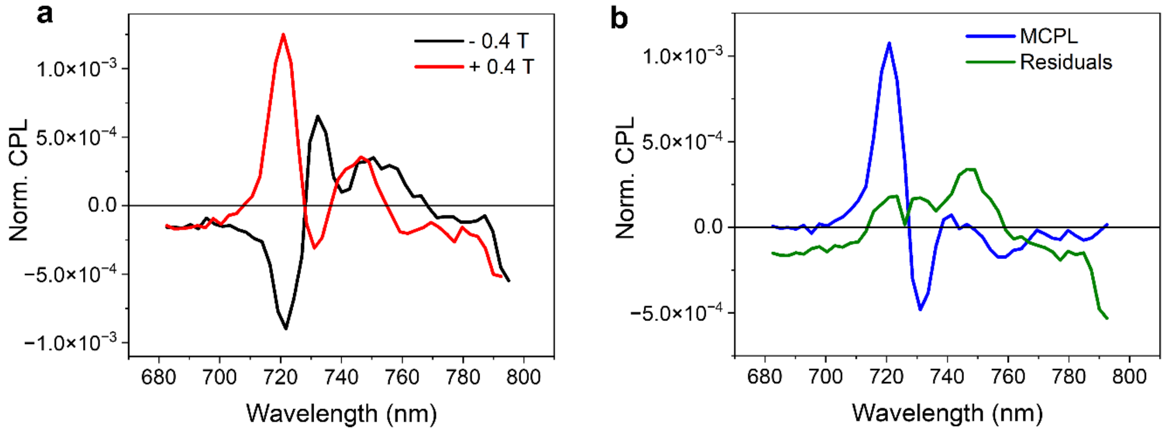

Figure S3. a. Normalized MCPL signals under +/-0.4 T; **b.** MCPL spectrum obtained by the semi-difference of the signals in **a** (see eq. 2 in the text), and the residuals obtained as the semi-sum of the signals.

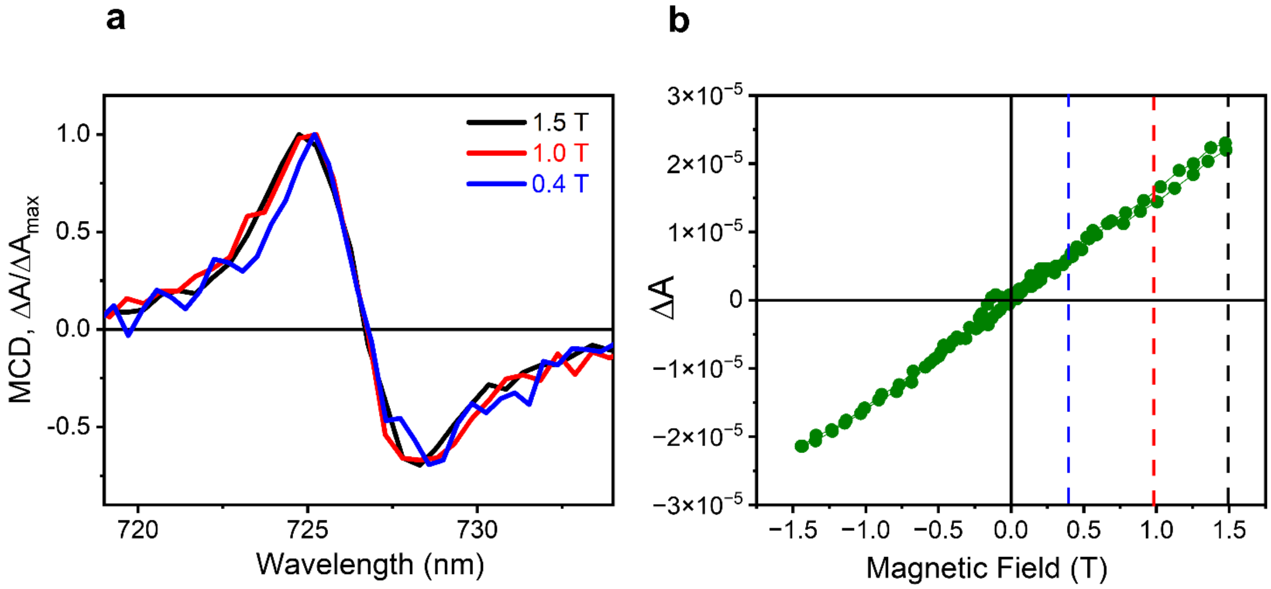

Figure S4. a. MCD spectrum of the most intense the spin-flip transition at different applied magnetic fields; **b.** MCD signal at 725 nm as a function of the applied magnetic field. The vertical lines in **b** show the applied field used to acquire the spectra in a.

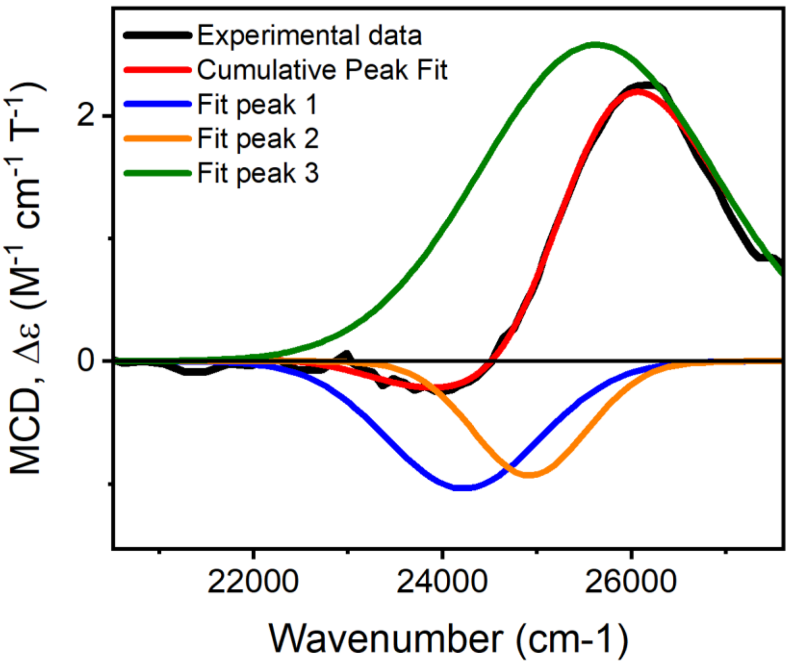

Figure S5. Phenomenological fitting of the low energy tail of the MCD spectrum.

Table S1. Values obtained from the fitting of the low energy tail of the MCD spectrum.

	Parameter[a]	Value	Standard Error
**Peak 1**	**E**	24220 cm^{-1}	3112 cm^{-1}
	**A**	-838	13
	**σ**	809 cm^{-1}	1186 cm^{-1}
**Peak 2**	**E**	24922 cm^{-1}	356 cm^{-1}
	**A**	-559	15
	**σ**	601 cm^{-1}	394 cm^{-1}
**Peak 3**	**E**	25630 cm^{-1}	795 cm^{-1}
	**A**	3175	19
	**σ**	1228 cm^{-1}	271 cm^{-1}

[a] E = energy, A = area of the Gaussian, σ = peak full width at half maximum.

According to Lenz *et al.*[3], in a D_2 geometry, the zero-field parameters D and E can be calculated taking into account the energies ΔE_i of the 3 components of the $^4A_2 \rightarrow {}^4T_2$ transitions (with $\Delta E_3 > \Delta E_2 > \Delta E_1$):

$$D = \frac{1}{2}\zeta^2\left(\frac{8}{\Delta E_1} - \frac{4}{\Delta E_2} - \frac{4}{\Delta E_3}\right) \quad \text{(eq S1)}$$

$$E = \frac{1}{2}\zeta^2\left(\frac{4}{\Delta E_2} - \frac{4}{\Delta E_3}\right) \quad \text{(eq S2)}$$

Where ζ is spin-orbit coupling constant of the free Cr(III) ion (273 cm^{-1}).[4] The ZFS can be calculated as:

$$ZFS = 2\sqrt{D^2 + 3E^2} \qquad \text{(eq S3)}$$

Analysis of MCPL data

According to the rigid shift model, the MCPL can be modelled by two equal, but opposite, bell-shaped functions displaced by the Zeeman splitting (Z), with respect to the barycenter energy (E). Such functions share the same parameters with the one used to fit the photoluminescence band (with the same bandwidth σ, peak area, etc.) and are obtained by simultaneously fitting the MCPL and the PL (Figure S6).

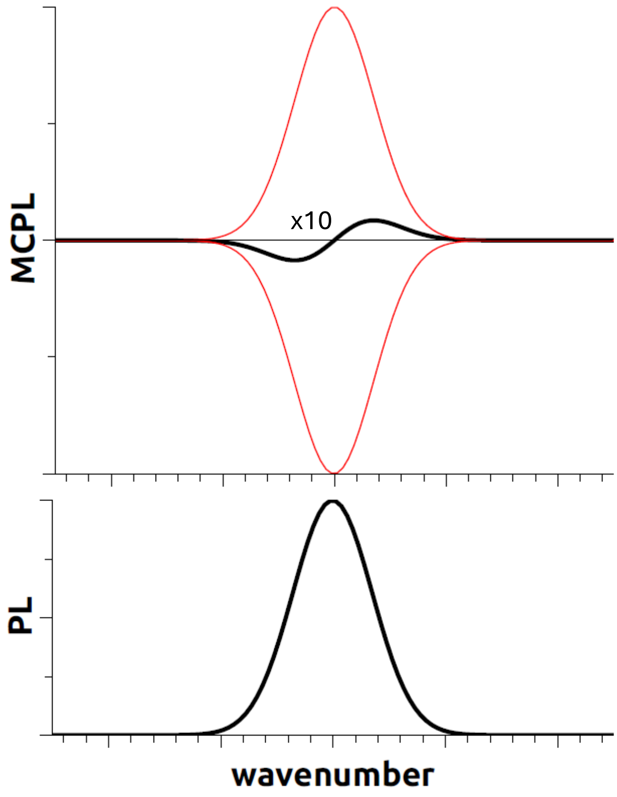

Figure S6. Illustrative example of the rigid-shift approximation applied to MCPL. The derivative shape (amplified 10 times for the sake of visualization) emerges from the partial cancellation of two opposite sign identical Gaussian functions translated by a dx (here σ/dx = 100)

Fitting functions

We chose pseudo-Voigt line shapes (eq S12) to fit the PL spectra, one for each of the two emissive transitions.

$$PL_i = A_i\left[\mu_i \frac{2}{\pi}\frac{\sigma_i}{4(x-E_i)^2+\sigma_i^2} + (1-\mu_i)\frac{\sqrt{4\log 2}}{\sqrt{\pi}\sigma_i} exp\left(\frac{-4\log 2\cdot(x-E_i)^2}{\sigma_i^2}\right)\right] \qquad \text{(eq S4)}$$

With $i = 1{,}2$, where A_i is the peak Area, σ_i the full width at half maximum, E_i is the energy centre of the PL peak, while μ_i is a shape parameter with values in the range from 0 to 1, with 1 representing a pure Lorentzian shape and 0 a pure Gaussian shape.

MCPL of each SF transition is modelled as the difference between two sets of peak functions (two positive and two negative) with the same parameters used in equation S12 to fit the PL spectrum, but shifted in energy by a factor ΔE_i. Each fitting function used to model MCPL is therefore:

$$MCPL_i = \pm\frac{1}{2}(A_i \pm dA_i)\left[\mu_i \frac{2}{\pi}\frac{\sigma_i}{4(x-E_i-\Delta \boldsymbol{E}_i)^2+\sigma_i^2} + (1-\mu_i)\frac{\sqrt{4\log 2}}{\sqrt{\pi}\sigma_i} exp\left(\frac{-4\log 2\cdot(x-E_i-\Delta \boldsymbol{E}_i)^2}{\sigma_i^2}\right)\right] \quad \text{(eq S5)}$$

where dAi is added as an empirical parameter accounting for the slight asymmetry of the MCPL derivative-like signal. ΔE_i is expressed using the energy of the ground state sublevels found through HFEPR analysis. In particular, for the 4 transitions depicted in Figure 4 (main text), $\Delta \boldsymbol{E}_i$ is reduced to the following expressions for $i = 1 - 4$:

$$|+\mathbf{1/2}> \rightarrow |-\widetilde{\mathbf{1/2}}>: \quad \Delta \boldsymbol{E}_1 = +\frac{\boldsymbol{Z}}{\mathbf{2}} - \mathbf{0.44}\ \boldsymbol{cm}^{-1} \quad \text{(eq S6)}$$

$$|-\mathbf{1/2}> \rightarrow |-\widetilde{\mathbf{3/2}}>: \quad \Delta \boldsymbol{E}_2 = -\frac{\boldsymbol{Z}}{\mathbf{2}} + \mathbf{1.03}\ \boldsymbol{cm}^{-1} \quad \text{(eq S7)}$$

$$|-\mathbf{1/2}> \rightarrow |+\widetilde{\mathbf{1/2}}>: \quad \Delta \boldsymbol{E}_3 = -\frac{\boldsymbol{Z}}{\mathbf{2}} - \mathbf{0.66}\ \boldsymbol{cm}^{-1} \quad \text{(eq S8)}$$

$$|+\mathbf{1/2}> \rightarrow |+\widetilde{\mathbf{3/2}}>: \quad \Delta \boldsymbol{E}_4 = +\frac{\boldsymbol{Z}}{\mathbf{2}} + \mathbf{0.07}\ \boldsymbol{cm}^{-1} \quad \text{(eq S9),}$$

Where Z is considered as: $Z = \mu_B g \mathrm{H}\Delta Ms = 0.36\ cm^{-1}$ (μ_B is the Bohr magneton and g the electron g-factor), for 0.4 T of applied field and $\Delta Ms = 1$, and is thus kept as a fixed parameter during the fitting. Note that the transitions associated to the energies in eq. give a positive sign contribution to MCPL, while those associated to eq. give a negative contribution (compare with Figure 4).

As it is very weak, in first approximation we neglect the low energy transition in the MCPL fitting functions.

Thus, equations S4 and S5 are used to fit simultaneously the PL and MCPL spectra, using a home-built matlab routine. In Figure S7 we show the fitting using different peak functions: Gaussian, Lorentz or Pseudo-Voigt functions, which is a linear combination of the first two functions. The parameters extracted from the fitting are reported in Table S2. We also report a fitting without the empirical parameter dA (dA = 0). The parameters obtained through the fitting are reported in Table S2. The pseudoVoigt function retraces better the PL and MCPL line shape, revealing a reasonable agreement with the experimental data, despite the significant approximation made in defining the fitting functions. On the other hand, similar line shapes are obtained with different peak functions, indicating the robustness of the fitting procedure.

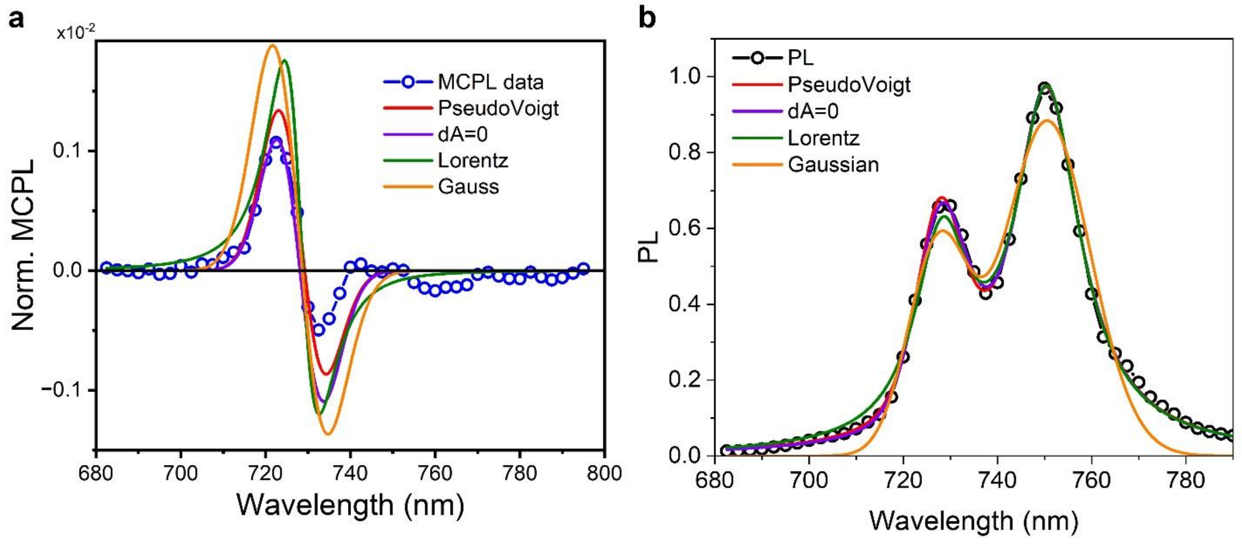

Figure S7. Fitting of normalized MCPL (a) and PL (b) spectra with different peak functions.

Table S2. Parameters extracted from MCPL and PL fitting of the high energy transition using equations S12-S13.

	**High energy transition**					**Low energy transition**			
	**A**	**σ** (cm^{-1})	**E** (cm^{-1})	**μ**	**dA**	**A**	**σ** (cm^{-1})	**E** (cm^{-1})	**μ**
**Pseudo-Voigt function**									
Param	0.011	247	13733	0.02	$1.2 \cdot 10^{-5}$	0.058	310	13324	1.00
Error	0.003	12	5	0.50	$0.6 \cdot 10^{-5}$	0.002	13	4	0.07
**dA fixed to 0**									
Param	0.011	247	13732	0.02	0*	0.058	309	13324	1.00
Error	0.004	14	5	0.54	-	0.002	15	4	0.08
**Lorentz function (μ=1)**									
Param	0.027	266	13729	1*	$1.9 \cdot 10^{-5}$	0.054	299	13320	1*
Error	0.006	29	11	-	$1.4 \cdot 10^{-5}$	0.004	20	6	-
**Gaussian function (μ=0)**									
Param	0.022	291	13740	0*	$1.6 \cdot 10^{-5}$	0.044	373	13319	0*
Error	0.010	44	32	-	$2.2 \cdot 10^{-5}$	0.006	41	16	-

* this parameter is kept fixed during the fitting

DC magnetometry

The *dc* magnetic properties of $[Cr(dqp)_2](PF_6)_3$ were studied in the 2-300 K temperature range with an applied magnetic field of 1000 Oe. The $\chi_M T$ vs T curve (χ_M is the molar magnetic susceptibility) is shown in Figure S7. The $\chi_M T$ value at room temperature of 1.90 $cm^3 mol^{-1}K$ is very close to expected value of 1.875 $cm^3mol^{-1}K$ for a Cr(III) ion with S = 3/2 if g = 2, which agrees with a quartet ground state ($^4A_2$). Upon cooling, the $\chi_M T$ product remains almost constant until about 10 K and then sharply decreases to reach a value of 1.78 $cm^3mol^{-1}K$ at 2 K.

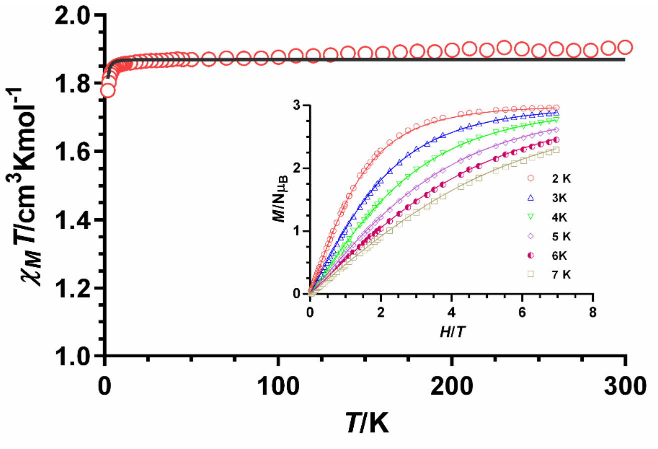

Figure S8. Temperature dependence of χ_{MT} (red circles) and field dependence of the magnetization at 2-7 K (inset). Solid lines represent the best fit to equation.

The field dependence of the magnetization of the Cr(III) compound in the 2-7 K temperature range and magnetic fields ranging from 0 to 7 T have been studied (Figure S7 inset). The magnetization values at 2 K and under the maximum applied field of 7 T of 2.97 Nβ match well with the theoretical saturation value of 3 Nβ expected for an isolated Cr(III) ion with g = 2 and S = 3/2.

The magnetic susceptibility and magnetization data were simultaneous fitted using the PHI program[5] with the ZFS spin Hamiltonian shown in equation 1.

$$\hat{H} = D\left[\hat{S}_z^2 - S(S+1)/3\right] + E(\hat{S}_x^2 - \hat{S}_y^2) + \mu_B \sum_{i=x,yz} g_i \vec{H}_i \hat{S}_i \quad \text{(eq S10)}$$

where the first and second terms account the axial and rhombic magnetic anisotropies, respectively, and the third term represents the Zeeman interaction. To improve the fit of the data and to avoid over-parameterization a term corresponding to the temperature independent paramagnetism (TIP) was included in the above Hamiltonian. It is worth noting that the low accuracy of the magnetic measurements for determining E and $|E/D|$ parameters prevent extracting very reliable ZFS parameters for the Cr(III) compound, particularly the sign of D (the fit of the data is the same with positive and negative D values) and the magnitude of E. In view of these considerations, E was fixed to zero in the fitting procedure. The best fit led to the following magnetic parameters: $|D|$ = 0.72 cm^{-1}, g = 1.99, TIP = 0.165×10^{-3} cm^3mol^{-1} and R = 2.5×10^{-7}. It is worth remarking that that magnitude of the D value extracted from the dc magnetic data of the compound is very similar to those experimentally and theoretically found for other Cr(III) complexes with slightly distorted octahedral coordination.[6,7]

HFEPR

HFEPR data analysis and simulation was accomplished using a software package SPIN by A. Ozarowski, freely available at: https://osf.io/z72tg/. The same software has a very useful option to calculate the mixing coefficients of the spin sublevels, and their corresponding energies, which is achieved by diagonalizing the spin Hamiltonian matrix containing the ZFS and Zeeman terms. The results of this procedure are shown in Tables S3 and S4.

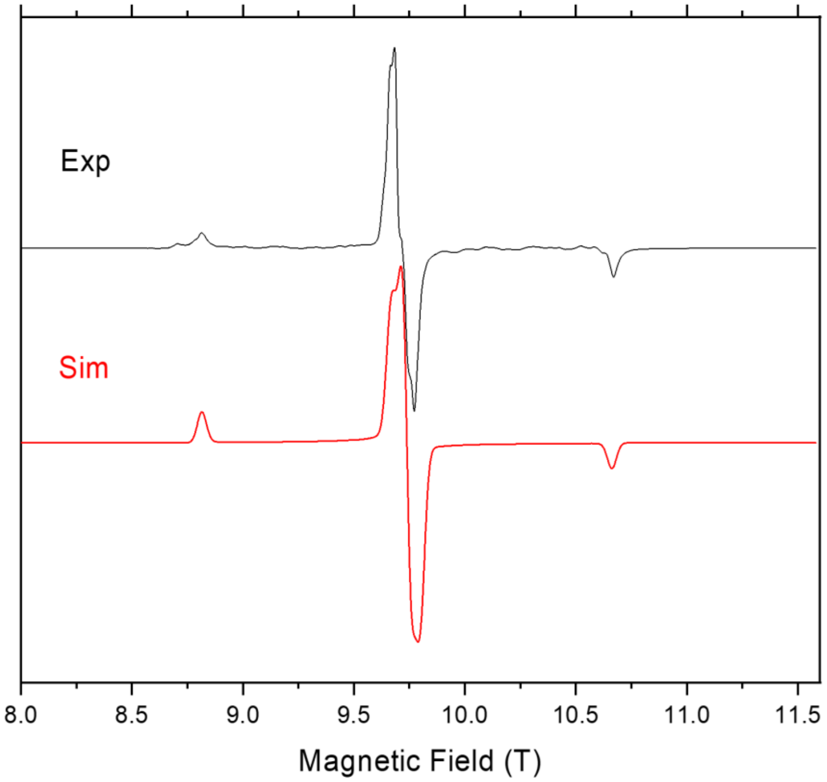

Figure S9. The allowed transitions in the EPR spectrum of of $[Cr(dqp)_2](PF_6)_3$ constrained as a pellet at 10 K and 270 GHz (black trace) accompanied by their simulation (red trace) using the following spin Hamiltonian parameters: S = 3/2, D = 0.43 cm^{-1}, E = 0.14 cm^{-1} (E/D = 0.325), g_x = 1.99, $g_{y,z}$ = 1.98. The simulation assumed a perfectly random orientation of the crystallites in space. The poorly resolved structure on the central line is due to either a deviation from the maximum rhombicity condition (E/D < 0.33) or slight g-anisotropy or both.

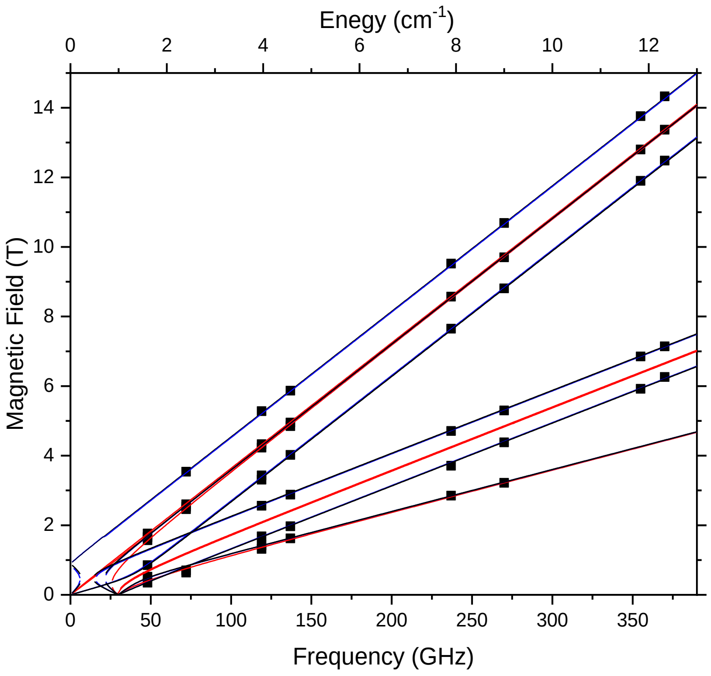

Figure S10. Field vs. frequency map of turning points (squares) in the EPR spectra accompanied by its simulations (curves)_using the following best-fitted spin Hamiltonian parameters: S = 3/2, D = –0.436 cm^{-1}, E = –0.134 cm^{-1} (E/D = 0.309), g_{iso} = 1.980. Red curves: magnetic field B_0 parallel to the x-axis of the ZFS tensor; blue: B_0 || y, ; blue: B_0 || z.

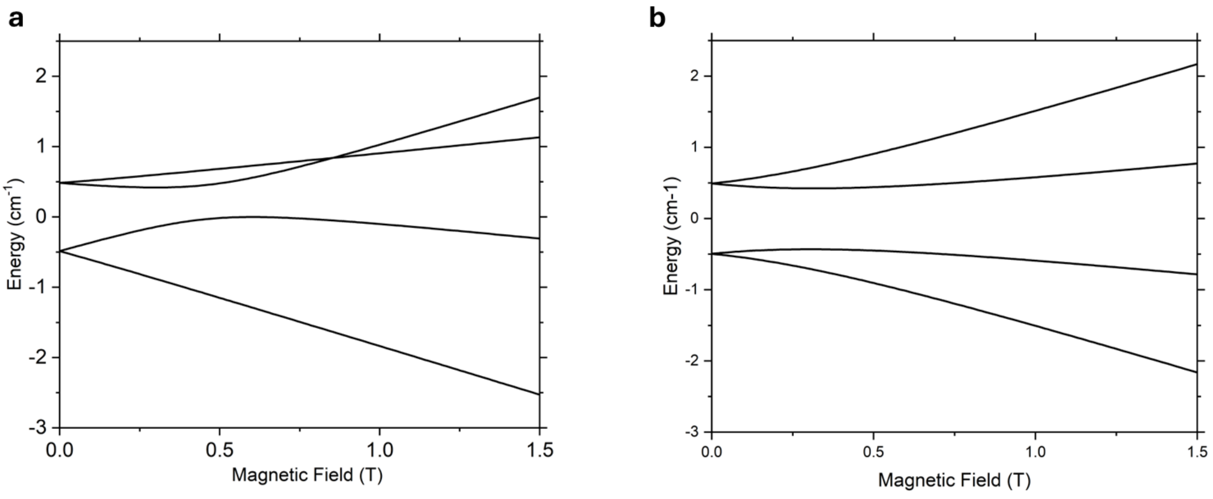

Figure S11. Representation of the HFEPR ground state energy levels for a S = 3/2 spin state for the low field region. a: B_0 || z, b: B_0 || x (for B_0 || y an almost equivalent situation to case a is obtained, albeit with an inverted level energy order).

Table S3. Energy levels of the spin sublevels of the $^4A_2$ ground state at 0.4 T (0 is the energy of the unsplit $^4A_2$ state) and mixing coefficients squared for $B_0 || z$ (an almost equivalent situation is obtained for $B_0 || y$). The first column indicates the main character of the state.

Mixed state	Energy / cm^{-1}	+3/2	+1/2	-1/2	-3/2
$\lvert\widetilde{+1/2}\rangle$	0.66026	0.00000	0.97784	0.00000	0.02215
$\lvert\widetilde{-1/2}\rangle$	0.44380	0.37843	0.00000	0.62157	0.00000
$\lvert\widetilde{+3/2}\rangle$	-0.07031	0.62157	0.00000	0.37843	0.00000
$\lvert\widetilde{-3/2}\rangle$	-1.03375	0.00000	0.022156	0.00000	0.97784

Table S4. Energy levels of the spin sublevels of the $^4A_2$ ground state at 0.4 T (0 is the energy of the unsplit $^4A_2$ state) and mixing coefficients squared for $B_0 || x$. The first column indicates the main character of the state.

Mixed state	Energy / cm^{-1}	+3/2	+1/2	-1/2	-3/2
$\lvert\widetilde{\pm 1/2}\rangle$	0.81387	0.00175	0.49825	0.49825	0.00175
$\lvert\widetilde{\pm 1/2}\rangle$	0.43793	0.15025	0.34975	0.34975	0.15025
$\lvert\widetilde{\pm 3/2}\rangle$	-0.44038	0.49825	0.00175	0.00175	0.49825
$\lvert\widetilde{\pm 3/2}\rangle$	-0.81142	0.34975	0.15025	0.15025	0.34975

Note that at high magnetic field the mixing is negligible and the order (from high to low energy) of the states is $|+3/2\rangle$, $|+1/2\rangle$, $|-1/2\rangle$, $|-3/2\rangle$.

References

1 J.-R. Jiménez, B. Doistau, C. M. Cruz, C. Besnard, J. M. Cuerva, A. G. Campaña and C. Piguet, *J. Am. Chem. Soc.*, 2019, **141**, 13244–13252.

2 A. K. Hassan, L. A. Pardi, J. Krzystek, A. Sienkiewicz, P. Goy, M. Rohrer and L.-C. Brunel, *J. Magn. Reson.*, 2000, **142**, 300–312.

3 S. Lenz, H. Bamberger, P. P. Hallmen, Y. Thiebes, S. Otto, K. Heinze and J. van Slageren, *Phys. Chem. Chem. Phys.*, 2019, **21**, 6976–6983.

4 P. E. Hoggard, *Zeitschrift für Naturforschung A*, 1981, **36**, 1276–1288.

5 N. F. Chilton, R. P. Anderson, L. D. Turner, A. Soncini and K. S. Murray, *J. Comput. Chem.*, 2013, **34**, 1164–1175.

6 G. Elbers, S. Remme and G. Lehmann, *Inorg. Chem.*, 1986, **25**, 896–897.

7 D. G. Liakos, D. Ganyushin and F. Neese, *Inorg. Chem.*, 2009, **48**, 10572–10580.
